# Variation in mandible development and its relationship to dependence on parents across burying beetles

**DOI:** 10.1002/ece3.4713

**Published:** 2018-11-21

**Authors:** Kyle M. Benowitz, Madeline E. Sparks, Elizabeth C. McKinney, Patricia J. Moore, Allen J. Moore

**Affiliations:** ^1^ Department of Entomology University of Arizona Tucson Arizona; ^2^ Department of Genetics University of Georgia Athens Georgia; ^3^ Department of Entomology University of Georgia Athens Georgia

**Keywords:** behavior/morphology correlations, heterochrony, mouthparts, *Nicrophorus*, parental care

## Abstract

**Background:**

In species with parental care, there is striking variation in offspring dependence at birth, ranging from feeding independence to complete dependency on parents for nutrition. Frequently, highly dependent offspring further evolve reductions or alterations of morphological traits that would otherwise promote self‐sufficiency. Here, we examine evidence for morphological evolution associated with dependence in burying beetles (*Nicrophorus* spp.), in which dependence upon parents appears to have several independent origins. In many species, precocial first instar larvae can survive without parenting, but several altricial species die at this stage on their own. We focused specifically on the mandibles, which are expected to be related to feeding ability and therefore independence from parents.

**Results:**

We find no evidence that the size of the mandible is related to dependence on parents. However, we do find a developmental and phylogenetic correlation between independence and the presence of serrations on the inner edge of the mandible. Mandibles of independent species bear serrations at hatching, whereas dependent species hatch with smooth mandibles, only developing serrations in the second instar when these larvae gain the ability to survive on their own. Phylogenetic evidence suggests that serrations coincide with independence repeatedly. We note a single exception to this trend, a beetle with a serrated mandible that cannot survive without parents. However, this exception occurs in a species that has recently evolved the loss of independence.

**Conclusions:**

We argue that the absence of mandible serrations occurs due to alternative selection pressures incurred in larvae dependent upon parents to survive. We suggest that this may have led to a variable function for mandibles, perhaps related to increased competitive ability among siblings or increased efficiency in receiving nutrition from parents. Furthermore, we propose that the phylogenetic pattern we see is consistent with the long‐held evolutionary hypothesis that evolutionary change in behavior and physiology precede morphological change.

## 1 BACKGROUND

One of the most striking aspects of systems displaying parental care is not the parenting itself, but rather the dependence displayed by offspring on their parents. All mammals and all but one taxa of birds (Gubernick & Klopfer, 1981; Jones & Birks, 1992) require some degree of direct, postnatal parental care to survive. Why has this dependence become so ubiquitous, when the ability to develop independently might serve as a strategy to deal with the contingency of parental death or abandonment, and what are its consequences for phenotypic evolution? Comparisons of altricial species, which require a relatively large parental investment to survive, and precocial species, which reach independence more rapidly (Starck & Ricklefs, 1998), have revealed several patterns. First, altricial species tend to display faster life‐history strategies than their precocial counterparts (Balon, 1981; Derrickson, 1992; Ricklefs, 1979). Thus, resources received from extended parenting may allow for more rapid growth. Second, despite more rapid overall growth, altricial species from a variety of animal taxa show delayed development of certain morphological characters, notably including eyes and external features such as fur, feathers, and cuticle (Derrickson, 1992; Nalepa et al., 2008; Nice, 1962; O'Reilly, Fenolio, Rania, & Wilkinson, 1998; Ricklefs, 1979; Werneburg, Laurin, Koyabu, & Sánchez‐Villagra, 2016). This pattern has been suggested to reflect the removal of selection pressures for offspring to produce traits related to their own independence (Alexander, 1990). However, altricial and precocial species represent a spectrum of dependent young, all of which require at least some parenting to survive. Here, we extend this type of comparison to an insect genus which exhibits discrete, between‐species variation in the ability to survive without parental care.

Burying beetles in the genus *Nicrophorus* provide a potential to investigate species making the transition from precocial to altricial young. Species in this genus can provide extensive and elaborate parental care by directly regurgitating partially digested carrion into the mouths of their begging offspring (Eggert & Müller, 1997; Scott, 1998), and offspring of all species benefit from receiving parental regurgitations (Lock, Smiseth, & Moore, 2004; Rauter & Moore, 2002). Parenting behavior is remarkably similar across species, to the extent that cross‐fostering between species is readily accomplished, with variable effects on fitness (Benowitz, Moody, & Moore, 2015; Capodeanu‐Nägler, de la Torre, Eggert, Sakaluk, & Steiger, 2018; Smith & Belk, 2018). However, although well developed, parenting is not necessarily obligatory and the extent that offspring depend on parental care varies (Smiseth, Darwell, & Moore, 2003; Trumbo, 1992). As well as variation in larval begging behavior (Smiseth et al., 2003), some larvae can survive from hatching without parents, while others will not survive past the first instar if parents are not present to feed (Capodeanu‐Nägler et al., 2016; Capodeanu‐Nägler, de la Torre, et al., 2018; Capodeanu‐Nägler, Eggert, Vogel, Sakaluk, & Steiger, 2017; Capodeanu‐Nägler, Prang, et al., 2018; Jarrett, Schrader, Rebar, Houslay, & Kilner, 2017). There are no major differences in feeding ecology between obligate and facultative care species (Capodeanu‐Nägler et al., 2016; Scott, 1998), making this an ideal system for comparative studies. The phylogeny of *Nicrophorus* and its closest relatives suggests that several independent transitions between obligate and facultative care have occurred, although the exact patterns of these transitions remain unclear (Jarrett et al., 2017; Sikes & Venables, 2013; Trumbo, Kon, & Sikes, 2001). Recently, Capodeanu‐Nägler, Prang, et al. (2018) present evidence that first instars of the obligate care species *N. orbicollis* can survive without parents if provided with oral secretions from parents and are able to consume flesh directly from a carcass. Thus, the primary mechanism for dependence on parents is not actually a lack of ability to self‐feed, but the physiology of the offspring and the requirement for nutrients provided in parental regurgitations. This physiological change has not led to strong coevolution of parental traits, as offspring of obligate care species can be successfully cross‐fostered by parents of facultative species (Benowitz et al., 2015).

Dependence on parenting in *Nicrophorus* displays opposing life‐history patterns from those observed in birds, fish, and mammals. Larger species, which develop more slowly as eggs and larvae (Benowitz et al., 2015), consistently display obligate care across the *Nicrophorus* phylogeny (Jarrett et al., 2017), whereas the opposite pattern is seen in vertebrates (Balon, 1981; Derrickson, 1992; Ricklefs, 1979). This raises the question of whether other morphological patterns associated with dependence may be different in burying beetles than in vertebrate parental systems. We focus our attention on the mandible, which is the primary feeding tool for many insects (Chapman & de Boer, 1995) and is frequently associated with variations in feeding performance (Bernays, 1998; Hochuli, 2001). Furthermore, experimental populations of third instar *N. vespilloides* larvae were recently found to evolve larger mandibles in the absence of parental care, suggesting a role for mandibles in self‐feeding ability (Jarrett et al., 2018). Specifically, we predicted that the mandibles of first instars of obligate care species would display markedly different morphology from all other larvae, as their evolution should be unconstrained by the need to self‐feed. We tested this prediction for mandible size, comparing length and allometry of mandible length between instars of the obligate *N. orbicollis* and the facultative *N. vespilloides*. During these initial investigations, we also noted a discrete variation in mandible structure, the presence/absence of serrations along the inner mandible edge. We therefore used a comparative approach to test our prediction for serrations by examining the prevalence of this trait in first instars across the *Nicrophorus* phylogeny, including four additional obligate care and three additional facultative care species.

## 2 METHODS

### 2.1 Sample collection

We collected *N. orbicollis* from Athens, GA and *N. vespilloides* from Cornwall, UK and maintained them at the University of Georgia at 25°C and a 14:10 light:dark cycle as described previously (Benowitz et al., 2015). We collected the facultative species *N. defodiens* and *N. tomentosus* from Oneida County, WI (Werner & Raffa, 2003) in September 2016 and maintained them under the same conditions. We collected the obligate *N. sayi* from Oneida County and Vilas County, WI (Werner & Raffa, 2003) in May 2017 and maintained them at 15°C and a 16:8 light:dark cycle, as this is a species requiring colder temperatures to breed (Benowitz, Amukamara, McKinney, & Moore, 2018). All species were maintained as outbred populations in the laboratory, and all were in the laboratory for more than one generation. We bred all beetles under their respective conditions by placing a male and a female in a plastic box (Pioneer Plastics, Dixon, KY, USA) and an 18‐ to 25‐g mouse carcass (RodentPro, Evansville, IN, USA). We collected first, second, and third instar larvae of *N. orbicollis* and *N. vespilloides*, along with first instar larvae of *N. sayi*,* N. defodiens*, and *N. tomentosus*. We preserved collected larvae in 75% ethanol and stored them at 4°C until dissection. We also searched the extensive Silphid collections at the Field Museum of Natural History in Chicago, IL for preserved first instar larvae and found single first instars preserved in 70% ethanol of six additional *Nicrophorus* species and two outgroup species. These species were the obligate species *N. americanus*,* N. investigator, N. marginatus*, the facultative species *N. mexicanus*, and two species with unknown behavior, *N. guttula* and *N. obscurus*. The outgroups were two species from the subfamily Silphinae that provide no parental care, *Necrophila americana* and an unidentified member of genus *Thanatophilus* from South Africa.

### 2.2 Analysis of mandible size

We examined mandible size in first, second, and thirds instars of *N. orbicollis* and *N. vespilloides* using light microscopy. We rinsed larvae twice in distilled water, removed the heads, and cleared the heads in 10% KOH. We then mounted each sample in KY Jelly (Reckitt Benckiser, Slough, UK), manually spread the mandibles away from the head capsule, and photographed them at 4x magnification using a Leica M80 stereomicroscope (Leica, Wetzlar, Germany). For allometric analysis, we measured mandible length from base to tip as well as vertical head capsule length using Leica Application Suite morphometric software (LAS V4.1). We averaged left and right mandibles for analysis as there was no difference between them. We used type I ANOVA to test the effects of species, head length, and their interaction on mandible length separately in each instar (*Nv*_1_ *n* = 21; *No*_1_ *n* = 35; *Nv*_2_ *n* = 23; *No*_2_ *n* = 29; *Nv*_3_ *n* = 20; *No*_3_ *n* = 18). We report results for the interaction term (head size and species were highly significant for all instars), which informs whether the species display allometric differences in mandible size. We performed statistical analysis in R 3.4.0 (R Core Team, 2018).

### 2.3 Analysis of mandible shape

We washed and cleared first and second instars of *N. orbicollis* and *N. vespilloides* as described above, as well as first instars of *N. sayi*,* N. defodiens*, and *N. tomentosus*, to closely examine qualitative variation in mandible shape across the five live collected species. We then separated the mandibles and mounted them in glycerol. When required, we removed mouthparts other than the mandibles to ensure good visualization of the mandible structure. Photographs of the cleared heads were taken using a Leica DNIRE2 inverted microscope (Leica, Wetzlar, Germany) with a Hamamatsu model C4742‐95 digital camera (Hamamatsu, Japan). To improve depth of field, a series of 4 to 7 images were stacked using Helicon Focus software (HeliconSoft, Kharkiv, Ukraine). Brightness and contrast of the overall images were adjusted using Adobe Photoshop CC (v. 2017.0.1) and, where needed, background debris was digitally removed from the image. For the eight museum collected samples, we cleared and mounted each larva as above, and imaged them using an OptixCam Summit K2 camera (Microscope.com, Roanoke, VA, USA) mounted to an Olympus SZ61 stereo microscope (Olympus, Tokyo, Japan). We then mapped first instar mandibular phenotype onto a partial phylogeny of burying beetles (Dobler & Müller, 2000; Sikes & Venables, 2013), including all species used in this study as well as other species previously identified as obligate or facultative (Jarrett et al., 2017).

## 3 RESULTS

**Figure 1:**
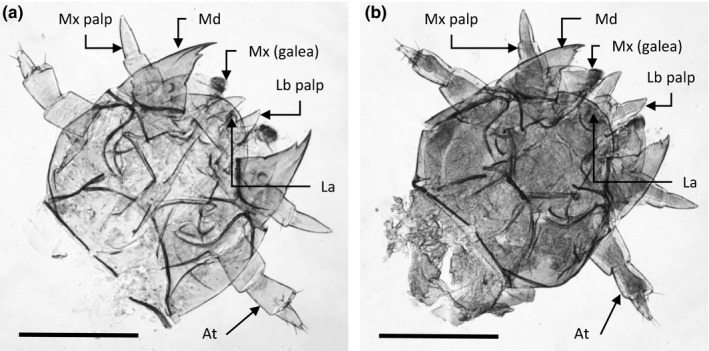
Head and appendages of first instar *Nicrophorus orbicollis* (a) and *Nicrophorus vespilloides* (b). Mouthparts and other appendages labeled (At: antenna; La: labrum, Lb: labium; Md: mandible; Mx: maxilla). Scale bars, 500 μm

**Figure 2:**
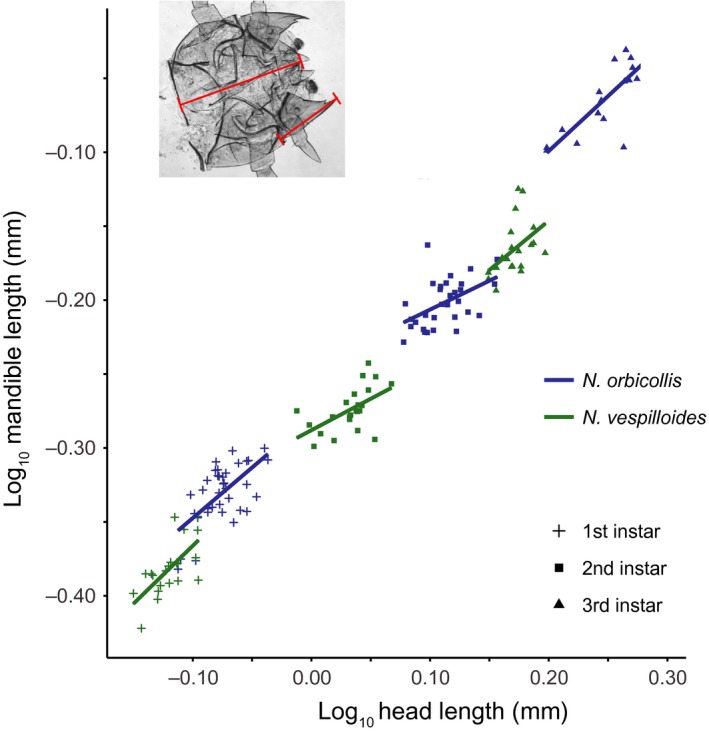
Allometric relationships between mandible length and head length in first, second, and third instar *Nicrophorus orbicollis* and *Nicrophorus vespilloides*. The image in the top left shows details on morphological landmarks used for measurement

We examined the size of larval mandibles in one altricial (obligate care) species, *N. orbicollis* (Figure 1a), and one precocial (facultative care) species, *N. vespilloides* (Figure 1b). Scaling relationships between mandible length and head length were hypoallometric in all three instars of both species, and especially so in second instars (*m_Nv_*_1_ = 0.784; *m_Nv_*_2_ = 0.784; *m_Nv_*_3_ = 0.668; *m_No_*_1_ = 0.683; *m_No_*_2_ = 0.391; *m_No_*_3_ = 0.744; Figure 2). Relative first instar mandible length was similar between the species, with *N. orbicollis* perhaps having slightly larger mandibles for a given body size (Figure 2). Furthermore, allometric relationships of mandible and head size between *N. orbicollis* and *N. vespilloides* did not differ in first (*F*_1,53_ = 0.16, *p* = 0.69), second (*F*_1,50_ = 0.03, *p* = 0.86), or third (*F*_1,35_ = 0.06, *p* = 0.81) instar larvae (Figure 2). Therefore, we find no evidence that *N. orbicollis* mouthparts develop at a slower rate than those of *N. vespilloides*.

**Figure 3:**
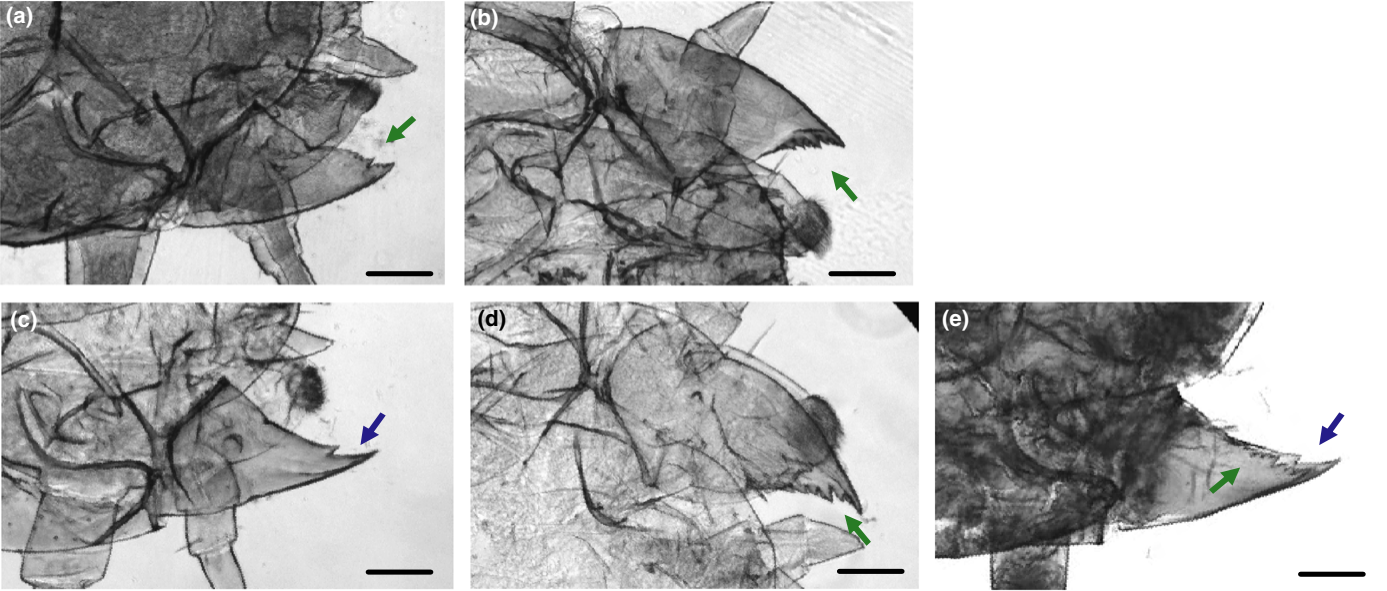
Mandible serrations in *Nicrophorus vespilloides* and *Nicrophorus orbicollis*. *N. vespilloides* first instar mandible showing the presence of serrations on the inner, cutting edge (a). *N. vespilloides* second instar with similar morphology (b). *N. orbicollis* first instar mandible lacking serrations (c). *N. orbicollis* second instar mandible with developed serrations above and below the initial large tooth (d). *N. orbicollis* late first instar mandible where developing second instar structure can be seen underneath the cuticle (e). Scale bars, 250 μm. Arrows indicate the location of the inner mandible edge, blue showing smooth, green showing serrated

We observed a considerable variation between the mandibles of *N. orbicollis* and *N. vespilloides* that was not captured by our morphometric measurements. Whereas the interior edge of *N. vespilloides* first and second instar mandibles contained numerous jagged serrations above and below the incisor (Figure 3a,b), *N. orbicollis* first instar mandibles were completely smooth (Figure 3c). Furthermore, when *N. orbicollis* molt into the second instar and gain the ability to survive independently, their mandibles concurrently develop serrations qualitatively similar to those seen in first and second instar *N. vespilloides* (Figure 3d). In late first instar larvae, collected just before molting, the serrated mandible can be seen developing underneath the cuticle of the smooth mandible (Figure 3e).

**Figure 4:**
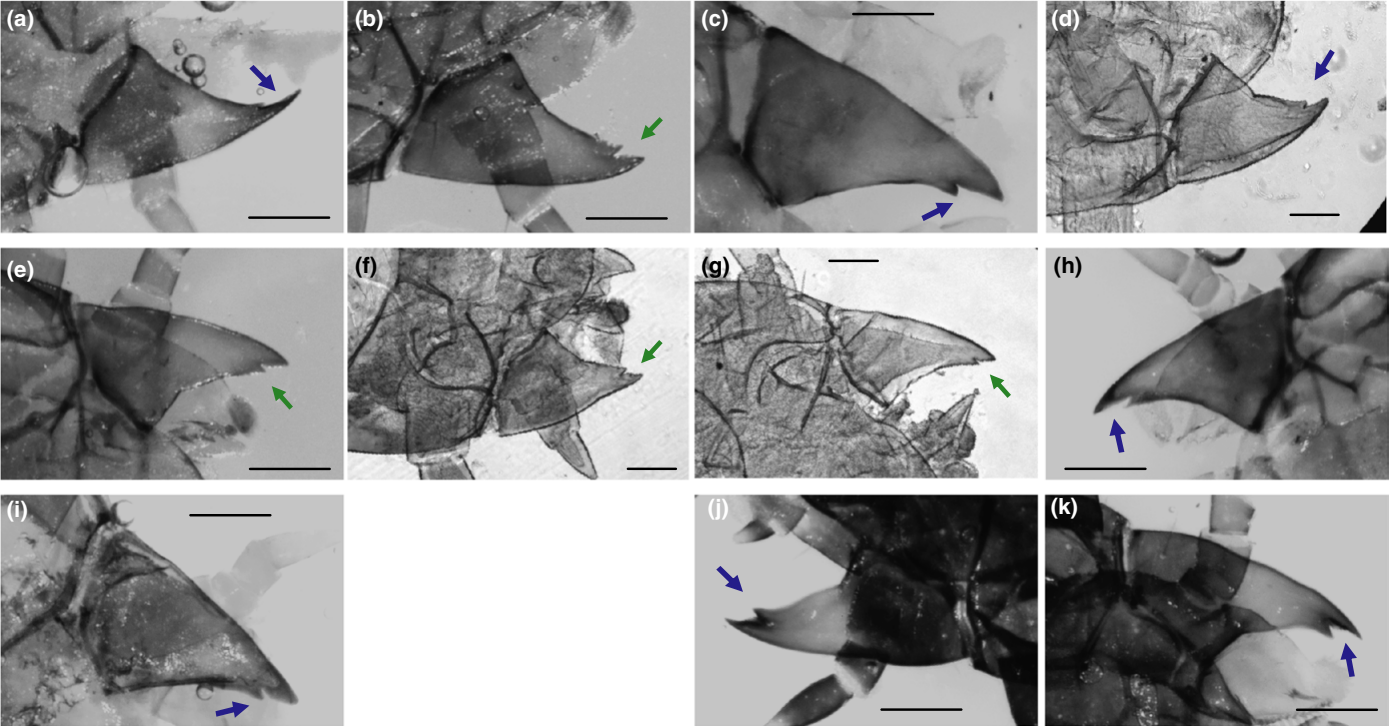
First instar mandible serrations across *Nicrophorus*. Species displaying obligate care (a–d): *N. marginatus* (a), *N. investigator* (b), *N. americanus* (c), *N. sayi* (d). Species displaying facultative care (e–g): *N. mexicanus* (e), *N. defodiens* (f), *N. tomentosus* (g). Species with no data on larval feeding (h, i): *N. guttula* (h), *N. obscurus* (i). Outgroups in the tribe Silphinae, with no parental care (j, k): *Necrophila americana* (j), unknown *Thanatophilus spp.* (k). Scale bars, 250 μm. Arrows indicate the location of the inner mandible edge, blue showing smooth, green showing serrated

**Figure 5:**
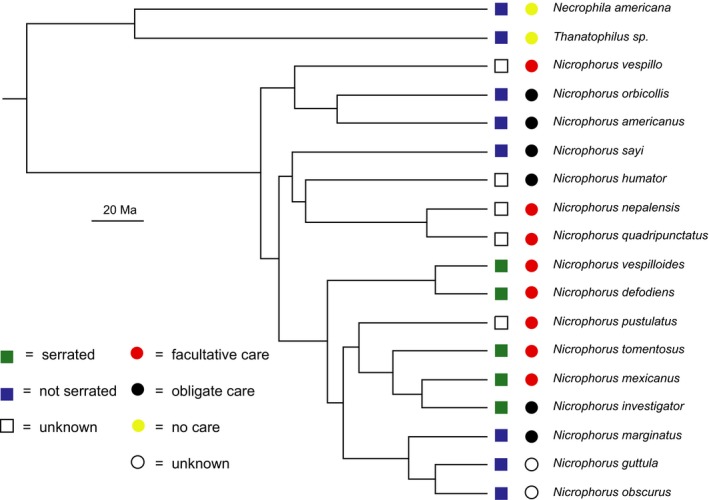
Phylogeny of *Nicrophorus* species for which parental dependence and/or serrations have been identified. Species relationships and divergence times from Dobler and Müller (2000) and Sikes and Venables (2013). Data on parental dependence from Jarrett et al. (2017) and references therein

Across burying beetles, we found that first instars in three of four additional obligate care species displayed smooth mandibles, the exception being *N. investigator* (Figure 4a–d), while all three additional facultative care species had serrated mandibles (Figure 4e–g). We also found smooth mandibles in two additional species for which no data on parental dependence has been reported (Figure 4h–i). First instars of two outgroups, which are carrion beetles that receive no parental care, had smooth mandibles (Figure 4j–k). A phylogeny of all species with either an ascribed mandible or behavioral phenotype is presented in Figure 5.

## 4 DISCUSSION

In many altricial systems, offspring evolve reduced size in structures that promote independence (Derrickson, 1992; Nalepa et al., 2008; Nice, 1962; O'Reilly et al., 1998; Ricklefs, 1979; Werneburg et al., 2016). Here, we expected independent larvae that might need to self‐feed to have larger mandibles, as mandible size is correlated to feeding ability is observed in other insects (Bernays, 1991) and larvae of the facultative species *N. vespilloides* have evolved larger mandibles during experimental adaptation to the absence of parental care (Jarrett et al., 2018). However, for the two species examined here, we found no such trend. Comparing the obligate *N. orbicollis* to *N. vespilloides*, there were no species differences in allometric relationships in any instar. Furthermore, mandibles of all instars in both species were hypoallometric, a pattern frequently interpreted to indicate that size is unrelated to functionality (Eberhard et al., 1998; Emlen, Warren, Johns, Dworkin, & Lavine, 2012; Shingleton & Frankino, 2013). Lastly, there did not appear to be much difference in relative mandible size for any instar; if anything, *N. orbicollis* mandibles were slightly larger for a given body size.

We did, however, find a strong association between dependence and the presence of serrations on the inner edge of the mandible, above and below the incisor tooth. These serrations are found in earlier descriptions of first instars of the facultative species *N. vespilloides (*Růžiĉka, 1992) and *N. mexicanus* (Palestrini, Barbero, Luzzatto, & Zucchelli, 1996), and third instars of all Nicrophorine (encompassing the genera *Nicrophorus* and *Ptomascopus*) species (Anderson, 1982). We found that these serrations were absent in first instar *N. orbicollis*, and that the mandible was instead completely smooth except for the incisor tooth. In addition, we found that *N. orbicollis* developed similar mandibular serrations during the second instar, corresponding to the developmental stage when larvae can survive without parental care. Serrations are related to the ability to consume meat across a variety of animal species (Godfrey, 1993) and have previously been implicated in a variety of cutting tasks in insects (Abler, 1992; Atallah, Teixeira, Salazar, Zaragoza, & Kopp, 2014; Roitberg et al., 2005). Thus, mandible serrations are likely to be useful for burying beetles in tearing flesh from carcasses, helping larvae to feed without the aid of parents.

Our phylogenetic analysis of first instar mandible serrations presented several intriguing trends. First, any potential relationship between serrations and dependence did not extend out to carrion beetles in general. The two outgroups examined here do not receive parental care and are thus completely independent, but do not display serrated mandibles. In fact, *Thanatophilus* species have smooth mandibles in all three larval instars (Daniel, Midgley, & Villet, 2017). In contrast, the nearest outgroup to burying beetles, *Ptomascopus morio*, which also does not exhibit parental offspring interactions but where parents prepare a carcass for larval consumption (Trumbo et al., 2001), has mandible serrations in at least the second (K. M. Benowitz unpubl. data) and third (Anderson, 1982) instars. Therefore, the presence of serrations altogether appears derived in the subfamily Nicrophorinae, but it is unclear why some larval carrion beetles but not others express this phenotype.

Within the *Nicrophorus* species for which we could obtain phenotypes for both traits, phylogenetic analysis largely pointed to a correlation between first instar serrations and dependence. Unfortunately, the directionality of transitions between obligate and facultative care is unclear, given that the majority of extant species have no relevant behavioral data (Jarrett et al., 2017) as well as the fact that ancestral and intermediate states are unclear. However, given the distribution of each behavioral state across the *Nicrophorus* phylogeny (Jarrett et al., 2017), it appears that they are not phylogenetically conserved and that independent evolutionary transitions have occurred a minimum of twice (if obligate care is ancestral) or four times (if facultative care is ancestral), with additional reversions likely in either case. Regardless of the true directionality, our data suggest that these transitions have generally coincided with transitions between smooth and serrated first instar mandibles. All measured facultative species, falling into two putative clades (*N. mexicanus/tomentosus* and *N. vespilloides/defodiens*), display serrated mandibles. Meanwhile, of four potentially independent obligate clades (*N. americanus/orbicollis*,* N. sayi/humator*,* N. investigator*, and *N. marginatus*), three display smooth mandibles. It is not yet clear whether the developmental correlation between serrations and independence observed in *N. orbicollis* extends to other species with smooth mandibles, as we have neither second instar morphological data for these species nor the behavioral data to determine whether independence commences in the second or third instar (Jarrett et al., 2017). However, in a coarse sense, this link is preserved in third instars, in which all species are independent (Jarrett et al., 2017; Trumbo, 1992) and display serrated mandibles (Anderson, 1982).

The one exception to the broad pattern in our first instar data is *N. investigator*, in which first instars display serrated mandibles despite requiring food from parents at birth. This finding rules out the possibility that serrations alone are responsible for the ability to self‐feed, and instead that they are necessary but not sufficient for survival without parents. Supporting this is recent data from Capodeanu‐Nägler, Prang, et al. (2018), who present two relevant results on *N. orbicollis*. First, they show that *N. orbicollis* first instar larvae do attempt to self‐feed but do so very inefficiently and thus cannot survive to the second instar on their own. Second, they demonstrate that experimentally providing larvae with oral fluids from parents allows larvae to survive and develop in the absence of parents themselves, whereas a smooth and easily consumable carcass paste does not. This strongly indicates that for obligate species, the inability to survive without parents is actually physiological, and related to specific factors provided by parents. Parental regurgitation in burying beetles appears to involve transfer of immunity as well as nutrition to offspring (Ziadie, Ebot‐Ojong, McKinney, & Moore, 2018).

Given the evidence that serrations and dependence on parents are not causally related, we hypothesize that in dependent species, relaxed selection (Lahti et al., 2009) frees mandibles to evolve beneficial alternative functions in larvae. Mandibular variations might provide a competitive advantage to larvae in two ways. First, they could increase the mechanical efficiency of being fed by a parent. Evidence for a similar phenomenon comes from parasitic rove beetles living within ant colonies, in which the evolution of reduced mandibular serrations is thought to be an adaptation to facilitate trophallaxis from workers (Akre & Hill, 1973; Parker & Grimaldi, 2014). Second, mandibular serrations could increase the likelihood of being fed by a parent by influencing sibling competition for parental attention. Larvae of some parasitoid wasps use sharpened mandibles as weapons in siblicide (Mayhew & van Alphen, 1999), and sharpened structures are used as weapons in other animal taxa (Caro, Graham, Stoner, & Flores, 2003; Frazzetta, 1988). *N. orbicollis* larvae have a size advantage when reared in smaller broods (Benowitz & Moore, 2016) and could therefore benefit from improved performance in intrafamilial combat.

Regardless of the specific selective mechanisms influencing the development of serrations, the question remains as to why only one studied species, *N. investigator*, does not display the association between these two traits. It is possible that the selection pressures favoring smooth mandibles in other obligate care species are simply absent in *N. investigator*, although there is nothing in the literature on this species to suggest it has any unique or unusual biological features, and as in other burying beetles large larvae display several advantages (Koulianos & Schwarz, 2014; Smith, 2002). Another, more intriguing explanation is that selection for smooth mandibles is present but has yet to result in observable phenotypic change. One notable observation about the *Nicrophorus* phylogeny is that of all species identified as obligate, only *N. investigator* displays evidence for recent evolution of this trait, as its nearest relatives display facultative care (Jarrett et al., 2017). This is consistent with the idea that morphologies may evolve more slowly than behaviors or physiologies (Blomberg, Garland, & Ives, 2003; Wcislo, 1989; West‐Eberhard, 1989), and thus optimal phenotypic combinations may take considerable time to arise. This may be especially true here because the relevant morphological change is specific to first instars, and therefore constrained by the need to maintain phenotypic stasis at other developmental stages (Moran, 1994).

This suggests a roadmap for how offspring can evolve in an environment where parenting is necessary and therefore ubiquitous. If parenting is dependable, offspring may experience relaxed selection on traits related to independence, which can lead to one of several outcomes: trait reduction, neutral trait persistence, or the evolution of novel function (Lahti et al., 2009). In a variety of systems studied to date, morphological evolution in altricial offspring is most consistent with a response of trait reduction (Derrickson, 1992; Nalepa et al., 2008; Nice, 1962; O'Reilly et al., 1998; Ricklefs, 1979; Werneburg et al., 2016). Although more data will be needed to completely unravel behavior/morphology relationships in burying beetles, the evidence presented here points to the evolution of a novel function in response to relaxed selection. We hypothesize that such a function might involve an increased efficiency in receiving food from parents or an improved ability to compete for parental attention. In this way, variable selection pressures across species may have promoted a unique and unexpected adaptive correlation between parental behavior, offspring physiology, and offspring morphology.

## CONFLICT OF INTERESTS

The authors declare they have no competing interests.

## AUTHORS' CONTRIBUTIONS

KMB, PJM, and AJM conceived and designed the study. KMB and ECM bred and collected live larvae. MES and PJM dissected and imaged live‐bred larvae. KMB obtained, dissected, and imaged larvae from museum collections. MES measured head capsule and mandible sizes. KMB analyzed the data. KMB, PJM, and AJM wrote the manuscript with input from all authors. All authors read and approved the final manuscript.

## DATA ACCESSIBILITY

Measurement data for *N. orbicollis* and *N. vespilloides* mandible and head capsule size are deposited in Dryad Digital Repository: https://doi.org/10.5061/dryad.6hp93cd. All other data are included in this article.

## ETHICS APPROVAL AND CONSENT TO PARTICIPATE

All institutional, national, and international guidelines involving the care and use of insects for research were followed.
